# Third-Party Disability for Significant Others of Individuals with Tinnitus: A Cross-Sectional Survey Design

**DOI:** 10.3390/audiolres13030033

**Published:** 2023-05-23

**Authors:** Eldré W. Beukes, Gerhard Andersson, Vinaya Manchaiah

**Affiliations:** 1 Vision and Hearing Sciences Research Centre, Anglia Ruskin University, Cambridge CB1 1PT, UK; 2 Virtual Hearing Lab, Collaborative Initiative between University of Colorado School of Medicine and University of Pretoria, Aurora, CO 80045, USA; 3 Department of Behavioral Sciences and Learning, Department of Biomedical and Clinical Sciences, Linköping University, 58183 Linköping, Sweden; 4 Department of Clinical Neuroscience, Karolinska Institute, 17177 Stockholm, Sweden; 5 Department of Otolaryngology—Head and Neck Surgery, University of Colorado School of Medicine, Aurora, CO 80045, USA; 6 UC Health Hearing and Balance, University of Colorado Hospital, Aurora, CO 80045, USA; 7 Department of Speech-Language Pathology and Audiology, University of Pretoria, Gauteng 0002, South Africa; 8 Department of Speech and Hearing, Manipal College of Health Professions, Manipal Academy of Higher Education, Manipal 576104, India

**Keywords:** significant others, third-party disability, tinnitus, tinnitus effects, tinnitus treatment, life effects, family members

## Abstract

There is currently increasing awareness of third-party disability, defined as the disability and functioning of a significant other (SO) due to a health condition of one of their family members. The effects of third-party disability on the SOs of individuals with tinnitus has received little attention. To address this knowledge gap, this study investigated third-party disability in the significant others (SOs) of individuals with tinnitus. A cross-sectional survey design included 194 pairs of individuals from the USA with tinnitus and their significant others. The SO sample completed the Consequences of Tinnitus on Significant Others Questionnaire (CTSOQ). Individuals with tinnitus completed standardized self-reported outcome measures for tinnitus severity, anxiety, depression, insomnia, hearing-related quality of life, tinnitus cognitions, hearing disability, and hyperacusis. The CTSOQ showed that 34 (18%) of the SOs were mildly impacted, 59 (30%) were significantly impacted, and 101 (52%) were severely impact. The clinical variables of tinnitus severity, anxiety, and hyperacusis in individuals with tinnitus were the best predictors of the impact of tinnitus on SOs. These results show that the SOs of individuals with tinnitus may experience third-party disability. The effect of the individual’s tinnitus on their SO may be greater when the individual with tinnitus has a higher level of tinnitus severity, anxiety, and hyperacusis.

## 1. Introduction

Tinnitus, defined as the perception of sound without a corresponding external sound source, has been associated with a range of physiological and psychological complaints, including insomnia, difficulty concentrating, depression, and anxiety [1]. Tinnitus can, hence, impact not only the individual, but also those living with them [2]. As the difficulties caused by tinnitus are not visible, as with physical difficulties, those with tinnitus often describe feeling that nobody understands the effects of tinnitus [3]. These effects include finding it difficult to maintain involvement in activities that they feel may exacerbate the tinnitus, such as attending certain social situations. Raising awareness of these difficulties associated with tinnitus is important for both the general public and those with tinnitus.

To increase understanding of the impact of health-related problems on functioning and disability, the World Health Organization developed an International Classification of Functioning, Disability and Health (ICF) framework [4]. Using the ICF has provided increased awareness of the wider negative impact disability can have on the individuals with the disability. The ICF relates disability to body functions and structure, activities, participation, and environmental factors. An individual’s level of functioning is viewed as a dynamic interaction between their health conditions, environmental factors, and personal factors on the ICF. According to the ICF, tinnitus is considered an aspect of body function (i.e., b2400—ringing in ears or tinnitus). The ICF has been used to demonstrate that tinnitus affects body functions, including emotional functions, sleep functions, hearing functions, sustaining attention, and energy levels [5]. Those severely affected by tinnitus reported changing aspects of their daily life to reduce exposure to sounds they believe may aggravate the tinnitus [6]. Some reduced participation in household tasks, family gatherings, or socializing in fear of negatively affecting the tinnitus. These lifestyle changes may, thus, have a direct impact on the significant others (SOs) of those with tinnitus. 

Recognition that health conditions, including hearing loss, affect SOs has led to the concept of third-party disability in the ICF framework [7]. Third-party disability refers to the difficulties faced by SOs due to their family member’s health condition [4]. SOs are often spouses or partners, but could also be family members or other individuals who have a close relationship to the individual with the disability. Due to most traditional rehabilitation efforts focusing solely on the person with the overt disability, many SOs may be “hidden victims”, including those with communication disorders [8,9,10,11,12]. 

Few studies have explored of the impact of tinnitus on SOs. Studies have generally examined the role of the spouse in moderating tinnitus experiences [13,14,15]. Another study reported that after those with tinnitus saw a professional, it was identified that family members generally had a greater understanding of tinnitus, felt tinnitus had less of an effect on the individual, and that those with tinnitus restricted their activities less often [16]. More recently, a qualitative study identified that the impact on SOs includes increased responsibility for household duties and childcare and a reduction in the frequency of attending social events, music concerts, and functions [17]. In some cases, this takes an emotional toll on SOs due to the increased stress and frustration they experience. This, in turn, can also negatively affect the relationship between significant others and the individuals with tinnitus [11]. Despite this detrimental impact on SOs, quantifying the resulting third-party disability for SOs of the with tinnitus has not previously been studied in a structured manner. This may be partially due to no tool being available to quantify the impact of tinnitus on SOs, although such measures exist for hearing loss, such as the Significant Other Scale for Hearing Disability (SOS-HEAR) [18]. To enable the measurement of third-party disability for tinnitus, the Consequences of Tinnitus on Significant Others Questionnaire (CTSOQ) was developed and validated as a self-report measure for SOs with tinnitus [19]. The aim of the present study was to identify the impact of tinnitus on SOs using the CTSOQ and to examine whether there are any predictors of this impact based on the disease characteristics of individuals with tinnitus.

## 2. Materials and Methods

### 2.1. Study Design

A cross-sectional survey design was used for data collection using dyads (i.e., individuals with tinnitus and their SOs). To ensure that best practice was followed, the Transparent Reporting of Evaluations with Nonrandomized Designs guidelines were used. Ethical approval was obtained prior to beginning the study from Lamar University (IRB-FY20-200). 

### 2.2. Participants

Participants consisted of pairs of individuals living in the USA, those with tinnitus and their self-selected SOs. Individuals with bothersome tinnitus were those who participated in trials of Internet-based cognitive behavioral therapy (ICBT) for tinnitus [20,21,22]. To be included, those with tinnitus needed a score of 25 or greater on the Tinnitus Functional Index [23], indicating significant difficulties with their tinnitus and the need for a tinnitus intervention. The participants, thus, represent those who find their tinnitus bothersome. Their task was to complete a series of outcome measures and consent to their SOs being involved in the study. Those who provided informed online consent could self-select SOs to whom to pass on the questionnaire link. SOs, in this context, were defined broadly to include those with a close relationship with the individuals with tinnitus (e.g., spouse, partner, parent, child, sibling, other family members, housemate, or close friend). The SOs had the opportunity to consider their involvement in the study. If they wished to participate, they needed to provide informed online consent before completing the questionnaire (see Supplementary Materials).

### 2.3. Data Collection

Data collection consisted of self-reported questionnaires provided electronically. Demographical information regarding each pair of participants was obtained, including gender, age, relationship of the SO to the person with tinnitus, whether the SO had tinnitus themselves, and whether they lived with the person with tinnitus. After this, the following self-reported outcome measures were completed.

#### 2.3.1. Outcome Measures for Individuals with Tinnitus

Clinical constructs measured included tinnitus severity as measured by the Tinnitus Functional Index (TFI) [23]; anxiety symptoms, measured by the Generalized Anxiety Disorder–7 (GAD-7) [24]; depression symptoms, measured by the Patient Health Questionnaire-9 (PHQ-9) [25]; insomnia, measured by the Insomnia Severity Index (ISI) [26]; general health-related quality of life (HRQoL) [27], measured using the EQ-5D-5L; tinnitus cognition, measured using the Tinnitus Cognitions Questionnaire (TCQ) [28]; and hearing disability and sound tolerance, measured using the Tinnitus and Hearing Survey (THS) [29]. The authors sought permission to use questionnaires that were not freely available. 

#### 2.3.2. Significant Others Outcome Measures

SOs completed only one questionnaire, the Consequences of Tinnitus on Significant Others Questionnaire (CTSOQ; Cronbach’s α 0.93). The CTSOQ is a structured questionnaire, developed and validated previously [19], and consists of 25 questions which focus on four sub-scales: (a) observations about the individual with tinnitus (10 questions); (b) personal impact (4 questions); (c) relationship impact (5 questions); and (d) providing support (6 questions) [19]. Each item is scored on a 5-point Likert scale, ranging from strongly disagree (0), disagree (1), sometimes (2), agree (3), and strongly agree (4). The scores are added to range between 0 to 100, with higher scores indicating substantial effects of tinnitus on SOs and their relationship. Scores between 0–25 indicate a mild impact, scores between 26–40 a significant impact, and scores of 41–100 a significant impact [19].

### 2.4. Data Analysis

The Statistical Package for Social Sciences [30] was used for statistical analyses. Descriptive statistics, including age, gender, and the relationship between the SO and the individual with tinnitus, were used to describe the sample characteristics for each group. Continuous variables were summarized with means and standard deviations. Categorical variables were described using frequencies and percentages. Where ordinal data (the individual Likert scale questions) were present, the median was reported. When the scores from the questions were combined (total scores), the mean scores were reported.

Correlations between CTSOQ score and each clinical variable were explored using a *p*-value of 0.05 for significance interpretation. Pearson’s product–moment correlation coefficients were used to estimate the strength of association between tinnitus severity and each variable. Correlation strength was categorized as very weak (0.00 to 0.19), weak (0.20 to 0.39), moderate (0.40 to 0.59), strong (0.60 to 0.79), or very strong (0.80 to 1.0). Hierarchical linear multiple regression models were utilized, with the impact of tinnitus on SOs (i.e., CTSOQ scores) as the dependent variable and the tinnitus-related clinical variables (clinical variables of tinnitus severity, anxiety, depression, and tinnitus cognition) as predictor variables. A *p*-value of 0.001 was used for significance interpretation, adjusted for multiple comparisons. The data met the assumptions of homogeneity of variance and the residuals were approximately normally distributed. There was no risk of multicollinearity, as indicated by the tolerance levels above 0.2 and variance inflation factor values below 10. Analysis of variance (ANOVA) and chi square testing were used to identify any group differences regarding baseline characteristics between those with different CTSOQ severity levels.

## 3. Results

There were 194 eligible pairs of participants (SOs and individuals with tinnitus). The age ranges were similar, at a mean of 55 (SD: 14) years for the SOs and 56 (SD: 12) years for the individuals with tinnitus, as seen in Table 1. The majority were living together (87%) and were partners (84%). When SOs were asked whether they experienced tinnitus, 18% reported having tinnitus themselves. The effects on individuals with tinnitus are seen in Table 1, indicating significant levels of tinnitus distress (55 out of 100).

### 3.1. Impact of Tinnitus on the Significant Others

**Figure 1:**
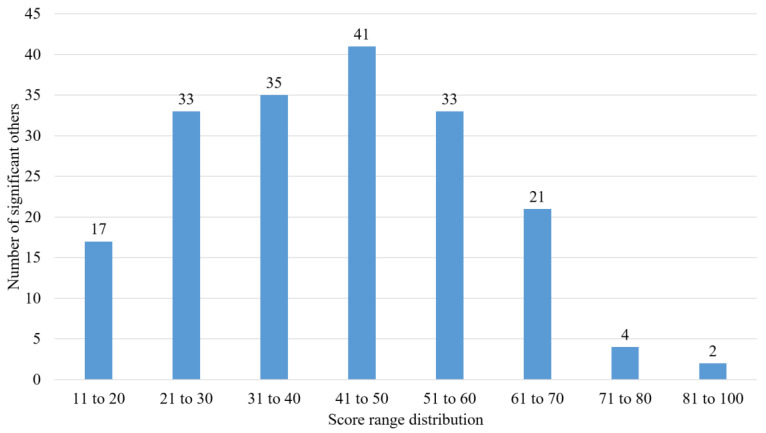
The impact of tinnitus on significant others using the score distribution of the Consequences of Tinnitus on Significant Others Questionnaire.

Total scores for the CTSOQ ranged widely, from 3 to 82, with a mean of 43 (SD: 16). The distribution of scores is shown in Figure 1, with the majority scoring between 21–60 on the CTSOQ. This indicated a mild impact for 34 (18%), a moderate impact for 59 (30%), and a significant impact for 101 (52%) the SOs. The median responses for each of the Likert Scale questions are shown in Table 2. These results indicated that the SOs were generally aware of the difficulties faced by the individual with tinnitus, but indicated that they did not know how to provide support to those with tinnitus. Although there was an impact on SOs, they were not always unduly affected in one area, but rather across all subscales (observations, personal and relationship impacts, and providing support). 

### 3.2. Associations between Tinnitus Severity and the Consequences on Significant Others

**Figure 2:**
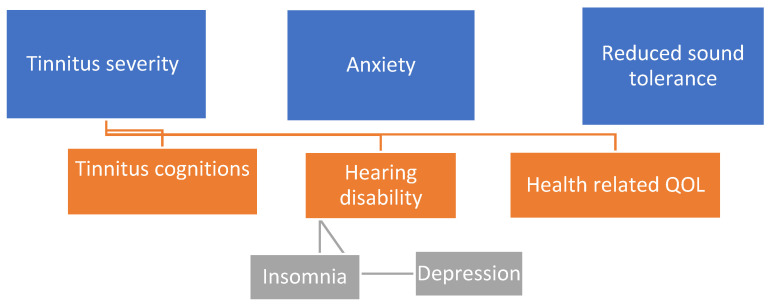
Hierarchy of the clinical predictors of the impact of tinnitus on significant others.

There was a moderate positive correlation between the consequences of tinnitus on SOs and the clinical variables of tinnitus severity, anxiety, depression, and tinnitus cognitions (see Table 3). There was a weak positive relationship between the consequences of tinnitus on SOs and the clinical variables of insomnia, health-related quality of life, hearing disability, and sound tolerance (see Table 3). All of these variables were, thus, included in a multiple regression model (see Table 3, Figure 2). The hierarchical linear multiple regression model indicated that the clinical variables from the individuals with tinnitus were able to predict the CTSOQ score of the SOs [*F* (10, 183) = 11.49, *p* < 0.001] and explained 39% of the variability of the CTSOQ score. The most significant predictors regarding the impact on SOs were tinnitus severity (*β* = 0.26, *p* = 0.02), anxiety (*β* = 0.26, *p* = 0.02) and reduced sound tolerance (*β* = 0.18, *p* = 0.02), as shown in the hierarchy in Table 3. 

## 4. Discussion

Third-party disability for SOs of individuals with tinnitus has not previously been studied using a structured approach. To address this knowledge gap, the CTSOQ was designed and validated to determine the effects of tinnitus on SOs [19]. This study was the first to quantify third-party disability for 194 SOs of individuals with tinnitus. The key findings are discussed below. 

### 4.1. The Consequences of Tinnitus on Significant Others

The impact of tinnitus was mild for 34 (18%), moderate for 59 (30%), and significant for 101 (52%) of the SOs. These findings suggest significant third-party disability for the majority of SOs of individuals with bothersome tinnitus. This impact was related to SOs identifying issues such as communication difficulties. Although participants with tinnitus attributed communication difficulties fully to their tinnitus, it is possible that hearing difficulties, which are often associated with having tinnitus, also contributed to these communication difficulties. Future studies need to establish the contributions of both. From many of the responses, it appeared as though both tinnitus and hearing-related difficulties contributed to this impact. The majority of scores were between 30 and 60 out of 100, although the score range varied widely between 2 and 89. These findings are comparable to the third-party disability noticed by SOs of individuals with hearing loss and vestibular problems [9,10,11,12]. This sample only included those with bothersome tinnitus who were seeking online psychological interventions [20,21,22]. It would be helpful to compare these findings with a general tinnitus population to identify whether the findings would be similar. It was encouraging that significant others noticed the impact that tinnitus has on individuals living with it, as indicated from the high scores on this subscale of the CTSOQ. The impact on relationships had the lowest score overall, which may be related to the SOs in this cohort appearing empathetic, acknowledging difficulties, and trying to provide support to the individuals with tinnitus. SOs indicated they wanted to learn more about ways to help their partners, suggesting that clinical interventions may be viewed favorably by both parties.

### 4.2. Associations between Tinnitus Severity and the Consequences on Significant Others

The clinical variables of tinnitus severity, anxiety, and hyperacusis were the best predictors of the impact of tinnitus on SOs. It was expected that the SOs of those with greater tinnitus severity would have increased third-party disability. This helps with triaging due to the heterogeneous nature of tinnitus, as not everyone is equally affected by having tinnitus [31,32]. Health professionals should be mindful that the SOs of individuals with higher levels of tinnitus severity and anxiety or the presence of hyperacusis may experience third-party disability. When identified, these SOs should be invited to attend tinnitus therapy sessions to help to increase their knowledge and understanding of tinnitus. The SOs should be monitored to determine whether attending these joint sessions decreases the third-party disability or whether further input is required. Furthermore, many other factors not investigated herein may have an impact on these results. The impact of marital satisfaction may be a confounding variable. It has previously been identified that poor marital cohesion is significantly associated with greater tinnitus severity, anxiety, depression, and mediated maladaptive coping [14,15]. 

### 4.3. Clinical Implications

These findings are important for identifying that third-party disability is present in the SOs of individuals with tinnitus. This has direct implications for clinical practice. Future models focusing on the wider context of the individual is necessary. It is possible that the third-party disability of the SO is an additional burden on the individual with tinnitus. Thus, measuring third-party disability routinely for the SOs of individuals with tinnitus would be prudent. Where third-party disability is identified, these SOs may benefit from involvement in the rehabilitation process [13]. Internet-based interventions can be one way to offer accessible and affordable management options for SOs, as they have been found to be effective for individuals with tinnitus [33,34]. There are examples of internet-based CBT for SOs in other areas [35,36,37], although none exist in the area of tinnitus. Nevertheless, this joint approach could benefit both the SO and the individual with tinnitus. More research should be conducted to identify effective joint care models, as no such intervention presently exists. This approach can be tailored depending on the individual’s needs. Encouraging SOs to attend appointments, support group meetings, and group sessions, and to make use of therapeutic support approaches, may help to increase their knowledge regarding tinnitus, and also to help their partners to feel supported. Informational counselling on the mechanisms and causes of tinnitus can help both those with tinnitus and their significant others develop a shared understanding. Individual sessions as well as group therapy approaches have been used in auditory rehabilitation program, including SOs [38]. When SOs were included in the rehabilitation programs, greater hearing handicap reduction was observed for individuals who had SOs attending group classes with them [39]. 

### 4.4. Limitations and Future Directions

Although this study provided us with some insights, these need to be considered within the context of this study. The participants represent the SOs of those with bothersome tinnitus who felt they required intervention to help them. They may, thus, not represent all individuals with tinnitus. Individuals who have more severe tinnitus are more likely to have passed on the questionnaire to their SOs. Further, SOs selecting to participate may be the ones noticing an effect causing a self-selection bias in the study sample. Although self-reported questionnaires were administered to those with tinnitus, they were not administered to SOs to determine their levels of anxiety and depression. Further studies should include SOs to collect these data. This study did not explore the dynamics of the relationships between individuals with tinnitus and their SOs. It may be that those who felt supported by their SOs were more likely to involve their SOs. Further bias may be introduced in some carers who were already being caring and supportive prior to receiving the guidelines for this study. Future studies should make an effort to include a more representative sample of SOs. In addition, further studies should be conducted to identify the effects of undertaking tinnitus intervention on SOs. 

## Figures and Tables

**Table 1:** Demographic profile of the pairs of significant others and individuals with tinnitus.

	Significant Others (SO)	Individuals with Tinnitus
Demographics N (%)/ Mean (SD) [Range]		
Mean age (standard deviation) [Range]	55 (14) [18–84]	56 (12) [21–81]
Gender		
Male Female Non-binary or other	100 (52%) 94 (48%) 0	77 (40%) 117 (60%) 0
Relationship		
Partner Parent Child Relative Friend	163 (84%) 3 (2%) 13 (7%) 9 (4%) 6 (3%)	
Living together n (%)		
Yes No	168 (87%) 26 (13%)	
Presence of self-reported tinnitus by the SO		
Yes No	34 (18%) 160 (83%)	
Clinical variables Mean (SD) (Range)		
Impact of tinnitus on SOs (CTSOQ) (range 0–100)	43 (16) [3 to 82]	
Tinnitus severity (TFI) (range 0–100)		55 (20) [7–96]
Anxiety (GAD-7) (range 0–21)		7 (5) [0–21]
Depression (PHQ-9) (range 0–27)		7 (5) [0–26]
Insomnia (ISI) (range 0–28)		11 (6) [0–27]
Health-related quality of life (EQ-5D-5L) (range 0–15)		8 (2) [5–18]
Health-related quality of life VAS (EQ-5D-5L VAS) (range 0–100)		76 (15) [20–100]
Tinnitus cognitions (range 0–104)		43 (16) [2–89]
Hearing disability (THS) (range 0–16)		7 (5) [0–16]
Sound tolerance (THS) (range 0–8)		1 (1) [0–4]

**Acronyms:** SOs = Significant others; GAD-7 = Generalized Anxiety Disorder–7; PHQ-9 = Patient Health Questionnaire–9; ISI = Insomnia Severity Index; EQ-5D-5L = General Health-Related Quality of Life; VAS = Visual Analog Scale; TCQ: Tinnitus Cognitions Questionnaire; THS = Tinnitus and Hearing Survey.

**Table 2:** Median responses to the Consequences of Tinnitus on Significant Others Questionnaire (CTSOQ).

Question	Median	Subscale Median
**Subscale: Observations about the individual with tinnitus**		**1.9**
Often worry about their tinnitus	2	
Have a poor quality of life	1	
Have difficulty concentrating or focusing their attention on what they are doing	2	
Have a low mood	2	
Are often anxious	2	
Have difficulty sleeping	2	
Have difficulty adjusting to experiencing tinnitus	2	
Are sensitive to certain sounds	3	
Participate in few activities or tasks	1	
Socialize less than before developing tinnitus	1	
**Subscale: Personal impact**		**1.3**
I experience a lot of stress	2	
My quality of life is poor	1	
There are more pressures on me due to the other person’s tinnitus	1	
I get annoyed with them	1	
**Subscale: Impact on the relationship**		**1.1**
We have difficulty communicating	2	
We do not socialize with other people as much as before tinnitus	1	
Our relationship has worsened	1	
We have been unable to focus on what is important in life	1	
**Subscale: Providing support (finding the following difficult):**		**1.7**
Showing sympathy	1	
Knowing how to help	2	
Encouraging the person with tinnitus	2	
Understanding what the effects of tinnitus are	2	
Understanding what tinnitus is	1	
Understanding why tinnitus is difficult to live with	1.5	

**Table Scoring:** The scores from the subscales are added together and the total score reported as a range between 0 to 100, with higher scores indicating substantial effects of tinnitus on SOs and their relationship. Scores between 0–25 indicate a mild impact, scores between 26–40 a moderate impact, and scores of 41–100 a significant impact [19].

**Table 3:** Correlations and hierarchical linear multiple regression model with impact of tinnitus on significant others (CTSOQ) as the dependent variable and tinnitus-related variables as predictor variables. Significant results are indicated by a *, representing *p* < 0.05.

Clinical Variables in Individual with Tinnitus	Pearson’s Correlation between the Significant Other Score and Tinnitus-Related Variables	Unstandardized Coefficient b (The Individual Contribution of Each Predictor to the Model), CI	Coefficient Standard Error Indicating the Extent These Values Vary across Each Sample SE b	Standardized Coefficients β	Whether the Predictor Is Making a Significant Contribution to the Model t-Value (*p*-Value Significance)
Constant		18.6 [−5.29 to 42.58]			*t* = 1.54, *p* = 0.13
Tinnitus severity (TFI)	*r* = 0.52, *p* < 0.001 *	0.21 [0.04 to 0.38]	0.09	0.26	*t* = 2.4, *p* = 0.02 *
Anxiety (GAD-7)	*r* = 0.48, *p* < 0.001 *	0.82 [0.15 to 1.48]	0.34	0.26	*t* = 2.4, *p* = 0.02 *
Depression (PHQ-9)	*r* = 0.49, *p* < 0.001	−0.21 [−1.0 to 0.60]	0.41	−0.07	*t* = −0.52, *p* = 0.61
Insomnia (ISI)	*r* = 0.40, *p* < 0.001 *	−0.08 [−0.60 to 0.44]	0.27	−0.03	*t* = −0.30, *p* = 0.77
Health-related quality of life (EQ-5Q-5L)	*r* = 0.38, *p* < 0.001 *	0.03 [−1.42 to 1.5]	0.73	0.003	*t* = 0.04, *p* = 0.97
Health-related quality of life VAS (EQ-5Q-5L VAS)	*r* = 0.33, *p* = 00.008 *	−0.03 [−0.25 to 0.18]	0.11	−0.03	*t* = −0.30, *p* = 0.76
Tinnitus cognitions (TCQ)	*r* = 0.45, *p* < 0.001 *	0.13 [−0.07 to 0.33]	0.10	0.12	*t* = 1.27, *p* = 0.21
Hearing disability (THS)	*r* = 0.23, *p* < 0.003 *	0.19 [−0.38 to 0.76]	0.29	0.05	*t* = 0.66, *p* = 0.51
Sound tolerance (THS)	*r* = 0.39, *p* < 0.001*	2.4 [0.46 to 4.35]	0.98	0.18	*t* = 2.45, *p* = 0.02 *

## Data Availability

The dataset is obtainable at http://doi.org/10.6084/m9.figshare.15062691 (accessed on 27 July 2021).

## Supplementary Materials

The following supporting information can be downloaded at: http://doi.org/10.6084/m9.figshare.15062691, (accessed on 27 July 2021).
